# The Open Door for Quackery

**Published:** 1892-02

**Authors:** 


### THE OPEN DOOR FOR QUACKERY.

Unfortunately, there does exist among medical men a certain class that refuses to be governed by the rules of professional propriety. Each of these is a law unto himself, and pursues his dubious occupation as his own morbid taste may dictate.

Often we notice that, with a boldness and presumption born of ignorance and unexcelled in any other class of men, he announces in the public prints that he is prepared to treat successfully every ailment in the wide range of human infirmity, from bee-sting to hydrocephalus, and from the indiscretions of ardent youth to the weaknesses of age.

Less often, with a rare and becoming modesty, he informs the public that his professional skill will be confined to the treatment of such medical and surgical commonplaces as cancer, or rupture, or catarrh, usually inserting the important and catchy promise, "no cure, no pay." Disease and death, sorrow and suffering would appear at least to be under absolute control. Even child-bearing woman, who has been laboring (no pun intended) for all these centuries under the primordial curse of bringing forth her young in sorrow, is now told with the pleasing and comforting assurance that her labors may be robbed of both discomfort and danger and be made as sweet as a dream of love in June.

Unfortunately, these apologies for physicians are far too numerous, but sadder still is the fact that there are many who repose a trustful confidence in the impudent statements and promises of these people, which they see in the papers. Along with these advertising frauds are to be classed also the patent medicine humbugs who detail to the public the universal curative properties of their worthless nostrums under high ministerial indorsement. Without conscience and without scruple this anomalous physician plays upon the fear and ignorance of his trusting patient and literally bleeds him for "all he is worth." That we are writing advisedly the following case will show: Not long since a young man, a newspaper reporter, came to our office for examination, with the history that he had been under the treatment of a rupture "specialist" for some time with no improvement in his condition. The rupture expert had clapped on a truss, of course, for which, with his treatment, he was making the modest charge of twenty-five dollars. On examination there was found a moderate degree of varicose enlargement of the spermatic veins of the left side. Nothing more, but this had probably been made worse by the pressure of his truss. This mistake was made either through ignorance or the intent to deceive. In either case the man that made it should not be allowed to practice medicine.

The trade of quackery, we all know, will take root and flourish wherever a suitable locality can be found, and, *a priori*, the most suitable locality is that one which offers the least obstacles to the conduct of the business.

In Georgia, we are sorry to say, the conditions are most favorable for the quack doctor; he enjoys the absolute liberty of the State, and pursues his shady occupation, unawed by shame or fear. In any community one of these men is a surplus; in Atlanta there is an overwhelming superabundance. Their faces and their cards, with an impudence and effrontery that is characteristic, appear daily in the columns of the public press. It matters not how dense their ignorance their professional skill seems to know no limitations. One of these applied to us recently, in great haste and excitement, for a quick emetic to enable him to throw up a half-drachm of sulphate of zinc which he had taken by mistake ! A medical contemporary, published in Atlanta, has already and truly announced, some weeks ago, that impostors are infesting the State, and that quackery is rampant in Georgia. The reason is to be found in the fact that Georgia makes no effort to keep them out. Here is a good opportunity for legislation. The State should exercise some discrimination as to what kind of persons shall practice physic within her limits. Instead of this, her doors are open wide, and he who desires may enter and practice his dangerous and shameless methods with none to molest or make afraid. If one wishes to run a dray, or keep a butcher-stall, or dispense peanuts and bananas on the street corners, he must get a license, but one—any one, that doesn't matter—can practice medicine for the asking. As long as this condition exists, you may depend upon it, our State will be the refuge for all the quacks and charlatans that are being driven from other States. The majority of States, we believe, have taken this matter in hand, and by their Examining Boards, or Boards of Health, are forcing these medical frauds and impostors to seek their dishonest livelihood elsewhere. The cause is just, and will finally prevail—even in Georgia.

But, at present, the door for quackery stands open wide—the more's the pity.

